# Bilateral Superficial Trigeminal Nerve Blocks are not More Effective than a Placebo in Abolishing Post-operative Headache Pain in Pituitary Transsphenoidal Neurosurgery: A Prospective, Randomized, Double-blinded Clinical Trial

**DOI:** 10.2174/1574887118666230227113217

**Published:** 2023-08-15

**Authors:** Una Srejic, Erik Litonius, Seema Gandhi, Pekka Talke, Oana Maties, Claas Siegmueller, Avic Magsaysay, Daniel Hasen, Sandeep Kunwar, Rahul Seth, Lizbeth Gibson, Philip Bickler

**Affiliations:** 1 Deparment of Anesthesiology and Pain Management, University of California, San Diego (UCSD) Medical Centre, San Diego, CA, USA;; 2 Department of Anesthesiology, Helsinki University Central Hospital, Intensive Care, Emergency Medicine and Pain, Helsinki, Finland;; 3 Department of Anesthesiology, University of California, San Francisco (UCSF) Medical Centre, San Francisco, CA, USA;; 4 Department of Family Comprehensive Cancer Center, University of California, San Francisco (UCSF) Medical Centre, San Francisco, CA, USA;; 5 Department of Neurosurgery, University of California, San Francisco (UCSF) Medical Centre, San Francisco, CA, USA;; 6 Department of Facial Plastic Surgery, Head and Neck Surgery, University of California, San Francisco (UCSF) Medical Centre, San Francisco, CA, USA

**Keywords:** Pituitary neurosurgery, superficial trigeminal nerve blocks, regional anesthesia of head and neck, supraorbital and infraorbital nerve block, migraine headache, supra-orbital V1

## Abstract

**Background:**

Pituitary neurosurgery executed *via* the transsphenoidal endonasal approach is commonly performed for pituitary adenomas. Reasons for prolonged hospital stay include postoperative headache and protracted nausea with or without vomiting. Bilateral superficial trigeminal nerve blocks of the supra-orbital V1 and infra-orbital V2 (SION) nerves performed intra-operatively as a regional anesthetic adjunct to general anesthesia were hypothesized to decrease 6 hours postoperative morphine PCA (patient-controlled analgesia) use by patients.

**Methods:**

Forty-nine patients, following induction of general anesthesia for their transsphenoidal surgery, were prospectively randomized in a double-blinded fashion to receive additional regional anesthesia as either a block (0.5% ropivacaine with epi 1:200,000) or placebo/sham (0.9% normal saline). The primary endpoint of the study was systemic morphine PCA opioid consumption by the two groups in the first 6-hours postoperatively. The secondary endpoints included (1) pain exposure experienced postoperatively, (2) incidence of postoperative nausea and vomiting, and (3) time to eligibility for PACU discharge.

**Results:**

Of the 49 patients that were enrolled, 3 patients were excluded due to protocol violations. Ultimately, there was no statistically significant difference between morphine PCA use in the 6 hours postoperatively between the block and placebo/sham groups. There was, however, a slight visual tendency in the block group for higher pain scores, morphine use p=0.046, and delayed PACU discharge. False discovery rate corrected comparisons at each time point and then revealed no statistically significant difference between the two groups. There were no differences between the two groups for secondary endpoints.

**Conclusion:**

It was found that a 6-hour postoperative headache after endoscopic trans-sphenoidal pituitary surgery likely has a more complicated mechanism involving more than the superficial trigeminovascular system and perhaps is neuro-modulated by other brain nuclei.

**Clinical Trial Registration Number:**

NCT04670614.

## 1 INTRODUCTION

Regional anesthesia combined with general anesthesia for minimally invasive procedures has become a common anesthetic technique. The goal of this approach is to reduce pain and promote rapid recovery [1].

If regional anesthesia facilitates the administration of less systemic pain medications, patients could benefit in several ways: (1) reduced postoperative discomfort and opioid use, (2) decreased postoperative nausea and vomiting, (3) shortened PACU (post-anesthesia care unit) stay time, and (4) shortened time to discharge from hospital. A more streamlined, shorter, and more pleasant hospital course for the patient could lead to increased patient satisfaction and potential economic gains for the hospital.

Pituitary tumor surgery is primarily executed through a minimally invasive approach through the nose and sinuses as an endoscopic procedure called the: “Trans-Sphenoidal Hypophysectomy”. More recently, it has been performed entirely endoscopically through the nose as the earlier modification, the “Caldwell Luc” approach, involved opening a bony window through a sub-labial oral (at the upper gum line under the top lip), leading to the maxillary sinus and then ultimately to the sellar cavity housing the pituitary tumor. Patients who undergo this endonasal endoscopic trans-sphenoidal pituitary surgery are usually same-day admission patients who, following their operation, stay in a hospital up to 24 hours postoperatively. Prolonged post-op admissions are often caused by persistent headaches or nausea with or without vomiting [2-5]. By administering regional anesthesia to the patient intra-operatively after induction of anesthesia, pain scores may be reduced [6], PONV decreased, and PACU stays shortened. Bilateral infra-orbital nerve blocks (V2 maxillary) have been shown to provide adequate pain control following trans-sphenoidal pituitary surgery such that systemic opioid use can be reduced or eliminated, and the patient discharged the earlier or same day of surgery [7]. The aim of our study was to investigate the effect of infra-orbital V2 and supra-orbital V1 nerve blocks to reduce morphine opioid use in the first 6 h post-procedure. Secondary endpoints of the study were: (1) pain exposure experienced by the patients postoperatively, (2) presence of PONV, (3) time to eligibility for PACU discharge, and (4) duration of hospital stay until discharge.

## 2 METHODS

Following Institutional Review Board approval at the University of California, San Francisco, USA (UCSF), study enrollment was offered to adult patients (male and female) (aged 18-65) having an ASA (American Society of Anesthesiologists) physical status I or II scheduled for same day admit endoscopic endo-nasal trans-sphenoidal hypophysectomy to be performed by a single surgeon (SK).

This is a prospective, randomized, double-blinded sham-controlled (preservative-free normal saline) regional anesthetic (supra-orbital and infra-orbital nerve (SION)) study to compare the systemic postoperative pain medication requirements in patients having trans-sphenoidal endoscopic, endo-nasal pituitary surgery under general anesthesia. The study protocol was performed in accordance with the relevant guidelines and is included in detail below. The primary endpoint was systemic morphine sulfate PCA (patient-controlled analgesia) opioid consumption by the 2 groups during the first 6 hours postoperatively (regional + general anesthesia vs. general anesthesia alone). The secondary endpoints included: (1) pain exposure experienced by the patients postoperatively, (2) incidence of PONV, (3) time to eligibility for PACU discharge, and (4) length of hospital stay/discharge. To our knowledge, this is the first published clinical trial examining the use of SION block to control postoperative pain after endonasal pituitary surgery. The results from the two groups were compared using an unpaired Student t-test. *p* < 0.05 was considered to indicate a statistically significant difference.

**Fig. (1):**
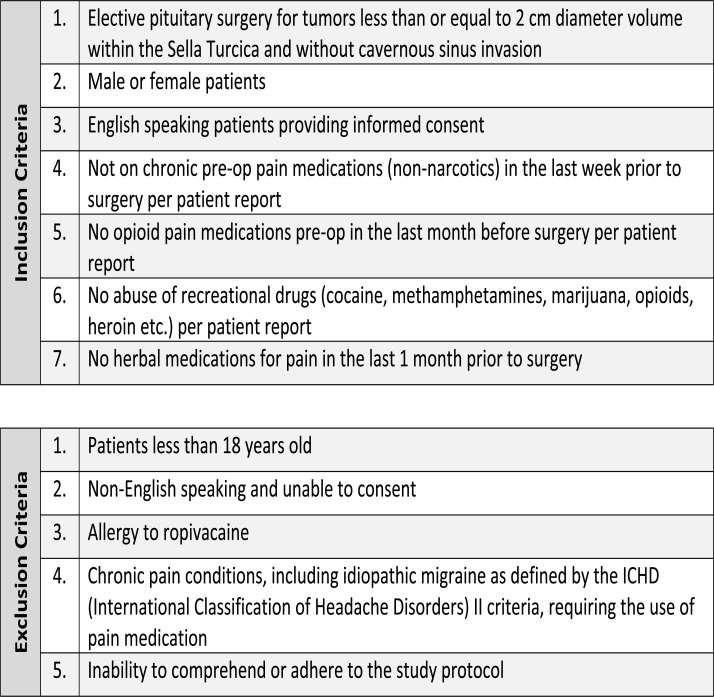
Pituitary pain study inclusion and exclusion criteria.

Additional inclusion criteria were: (1) elective pituitary surgery for tumors less than or equal to 2 cm diameter within the sella turcica and without cavernous sinus invasion, (2) both male or female patients, (3) English-speaking patients providing informed consent, (4) not on chronic pre-op pain medications (non-narcotics) in the last week prior to surgery as per the patient report, (5) no opioid pain medications pre-op in the last month before surgery as per the patient report, (6) no abuse of recreational drugs (cocaine, methampheta mines, marijuana, opioids, heroin, *etc*.) as per the patient report, and (7) no herbal medications for pain in the last 1 month prior to surgery as per the patient report. Urine or blood testing was not used to confirm opioid use preoperatively. Exclusion criteria included: (1) patients less than 18 yo, (2) non-English speaking and unable to consent, (3) known allergy to ropivacaine, (4) chronic pain conditions, including idiopathic migraine as defined by the ICHD (International Classification of Headache Disorders) II criteria, requiring the use of pain medications, or (5) inability to comprehend or adhere to the study protocol (Fig. **1**).

**Fig. (2):**
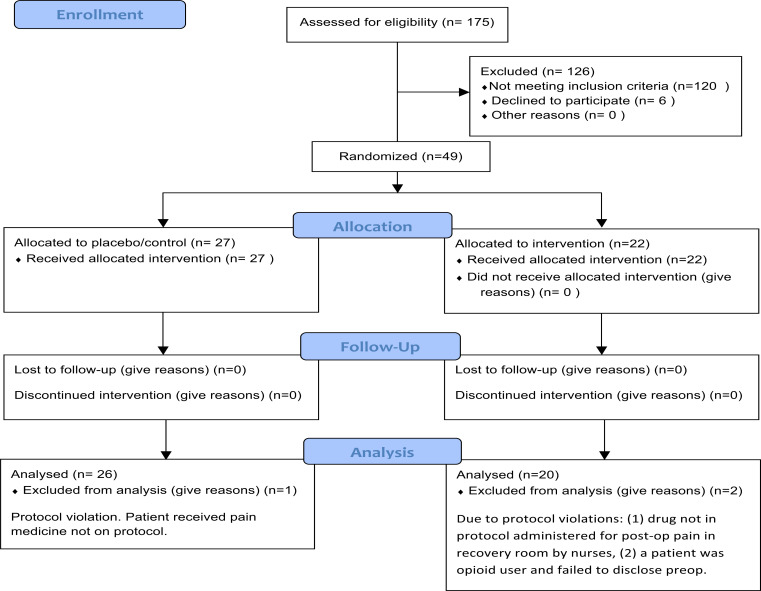
CONSORT flow diagram of participants. CONSORT indicates consolidated standards of reporting trials.

After written informed consent, the subjects were randomly assigned to 1 of 2 groups using randomization envelopes in 3 blocks of 20. A “triple mask design” was used: the subjects, healthcare providers (including the anesthesiologist in the operating room and anesthesiologist administering the SION blocks), randomization consent nurse, and data collection nurse, as well as the statistician, were all blinded to the treatment groups assigned (Fig. **2**).

### 2.1 Intra-operative Block Protocol

The subjects assigned to block group (B) received SION blocks with 0.5% ropivacaine with epinephrine 1:200 000 following induction of general anesthesia, and the placebo group (P) received the same volume SION blocks using sterile, preservative-free normal saline. Following general anesthetic induction and endotracheal intubation, supra-orbital and infra-orbital (SION) injections with study medication were performed. Supra-orbital nerve blocks were performed bilaterally using 3 cc (on each side for a total of 6 cc) of the study solution. A percutaneous technique was used to block both bilateral supra-orbital and supra-trochlear nerves. A 27 gauge, 1.5 needle attached to a 10-cc syringe was used. Then, the infra-orbital nerves were blocked intra-orally following retraction of the upper lip and using a 27-gauge, 2.54 cm needle attached to a 10-cc syringe inserted anterior to the ipsilateral canine tooth that runs along the maxilla. Here, 3 cc of the study drug was placed bilaterally for a total of 6 cc. The needles were directed superiorly toward the infra-orbital foramen on the ipsilateral side, as was described in the “Lynch” method [8]. Correct needle placement was confirmed by palpation of the needle tip near the infra-orbital foramen and by a negative aspiration for blood. A wheel of the study drug was palpated next to the nose under the inferior orbital rim. As mentioned earlier, the study drug was prepared by an independent physician who was not involved in the data acquisition, operating room care of the patient, or placement of the block. Thus, a total of 12 cc of the study solution was placed per patient in the distribution of the supra-orbital branches of V1 (Trigeminal sensory nerve) and infra-orbital nerve V2 (Trigeminal sensory nerve).

### 2.2 Intra-operative Anesthetic

Following pre-medication with midazolam 1-2 mg IV, anesthesia was induced with lidocaine 1 mg/kg IV, propofol 1-2 mg/kg IV, fentanyl 1 mcg/kg IV, and rocuronium 0.6 mg/kg IV.

The surgeon then administered standard topical intranasal anesthesia with lidocaine and epi-soaked pledgets on the inner nasal mucosa. Less than 10 cc of 1% lidocaine with epi 1:200 000 was used to ensure hemostasis. Access to the pituitary gland was obtained transnasally with an endoscope *via* the sphenoid sinus and then via the sellar floor. Following removal of the sellar floor, the dura was opened, and the pituitary tumor was resected using a series of micro-dissectors and curettes. CSF leaks were then repaired, and fat was placed in the free intra-sellar space. The sellar floor was repaired, and the sphenoid sinus and nostrils were packed temporarily to close the sellar space. We removed the nasal packing just prior to extubation.

Following induction of anesthesia, the local injection study block drug/placebo was administered, and the patients were maintained on a balanced anesthetic technique using intravenous agents, inhaled gas, and a neuro-muscular relaxant. Desflurane in O_2_/air mixture with 80% inspired O2 was administered to provide anesthesia and maintain the blood pressure within 20% of baseline. Fentanyl was infused at 1 mcg/kg/hr from induction of anesthesia to the end of surgery. Dexamethasone 10 mg IV or hydrocortisone 100 mg IV was administered at the beginning of surgery as directed by the surgeon. An end-tidal CO_2_ of 30-35 mmHg was maintained. The patients were relaxed with rocuronium as needed to maintain 1-2 twitches by an NMT (neuro-muscular twitch) monitor. On emergence from anesthesia, ondansetron 4 mg IV was administered, and residual muscle relaxation was reversed with neostigmine 0.07 mg/kg and glycopyrrolate 0.01 mg/kg IV.

Post-operatively, a morphine PCA (1mg IV every 6 min lock-out time) to a max of 10 mg IV morphine sulfate per hour, without a basal rate, was administered. For pain ranked at > 4/10, rescue pain medicine of morphine 1 mg IV was administered by the PACU recovery room nurses every 5-10 min until the pain was < 4/10 or acceptable to the patient. Blood pressure was maintained with a systolic BP < 140 mmHg using (labetalol, esmolol, and nicardipine). Following intraoperative treatment of nausea with ondansetron and dexamethasone/hydrocortisone, the following escalating rescue algorithm was used for PONV:

1) Additional ondansetron 4 mg IV for 1 dose, then

2) Prochlorperazine 5-10 mg IV, then

3) Scopolamine patch 1.5 mg topical [9].

The primary outcome of the study was to compare the total amount of morphine (in mg) used postoperatively (for 6 hours) in the 2 study groups from T0-T6 time from PACU entry (T0) to 6 hours post PACU entry (T6). Study data were collected for 5 data time points: T0 (PACU entry), T1, T2, T4, and T6 (1,2,4,6 hours after PACU entry, respectively). Pain scores (P0-P6) were collected using the Wong-Baker FACES Pain Rating Scale at times T0-T6 in the scale (0-10/10), with 10/10 being the worst imaginable pain. Nausea score N0-N6 was collected using the Baxter retching faces (BARF) nausea scale (0-10/10), with 10/10 being the worst nausea imaginable with or without vomiting. Other secondary data collected included Tr (total time in PACU from admission to “eligibility” for PACU discharge using the UCSF Modified Aldrete Score), Th (total time from surgery start to eligibility for home discharge), and Tn (time from surgery start to last dose of narcotics). Modified UCSF Aldrete PACU score in PACU assesses individual scores for vital signs, activity, mental status, pain, nausea, blood loss, and intake/output. At one-week post-op, patients were contacted by phone and asked about potential study complications.

A research assistant collected all study data and was blinded to the assigned treatment group.

## 3 STATISTICS

From Jan 1^st^, 2017 to Dec 31^st^, 2018, 175 patients were screened for the study using the inclusion and exclusion criteria (Fig. **1**). Based on clinical experience, we considered an effect size of 0.5 to be clinically significant. With this assumption, 64 patients were randomized into two equal groups for a statistical power of 0.8 for the main outcome. Of those, 49 patients were enrolled and randomized into the study. Although the original sample size was estimated at 64 patients, study enrollment was stopped after 49 subjects for administrative reasons.

Demographics and opioid consumption results are presented as means with 95% confidence intervals. Baseline variables presented in Table **1** report Absolute Standardized Differences defined as differences in means or proportions divided by the standard deviation (SD) and are used to assess the imbalance between the 2 groups. Established guidelines that interpret Standardized Differences describe the magnitude of the difference as: 0.2 = small, 0.5 = medium, and 0.8 = large. Comparisons between groups were performed with unpaired t-tests, Mann-Whitney u-tests, and Fisher’s exact test as appropriate. Parametric data are presented as means (95% confidence interval 95% CI), while nonparametric data are presented as medians (95% CI). Opioid consumption, pain exposure, and incidence of nausea over time were analyzed with a repeated measure mixed effects model. Groups were compared post hoc at each time point, correcting for multiple comparisons using the two-stage linear step-up procedure of Benjamini, Krieger, and Yekutieli to control the false discovery rate. Results with a *p* < 0.05 or a false discovery rate q < 0.05 were considered statistically significant. All analyses were performed using Prism 8.4.2 for MacOS (GraphPad Software, Inc, La Jolla, CA, USA).

The False Discovery Rate correction for multiple comparisons was chosen in order to preserve greater statistical power compared to the more often used Bonferroni correction (according to Glickman et al., false discovery rate control is a recommended alternative to Bonferroni-type adjustments in health studies, Journal of Clinical Epidemiology, 67(2014) 850-857).

## 4 RESULTS

A total of 49 patients were randomized. Three of the randomized patients subsequently had to be disqualified from the study due to protocol violations. There was no statistically significant difference between groups in demographic variables (Table **1**). Baseline variables reported as standardized differences were not substantially different between the 2 groups. They showed a possible small to a medium imbalance between the 2 groups (0.2-0.48). Height, weight, and BMI, which were more clinically relevant (as pain medication dosing was based on weight), showed a small or approximately 0.2 standardized difference.

**Fig. (3):**
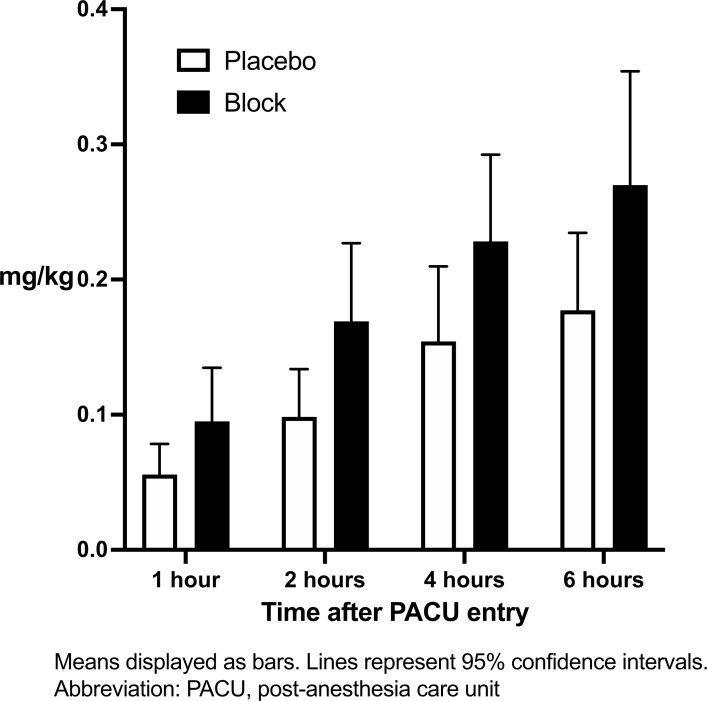
Cumulative post-operative intravenous morphine consumption in mg/kg in the PACU over 6 hours.

The median (95% CI) total postoperative morphine dose was 0.21 mg/kg (0.19 - 0.35 mg/kg) in the block group and 0.21 mg/kg (0.12 - 0.23 mg/kg) in the placebo group (*p* = 0.22). As analyzed over time (Fig. **3**), there was a significant difference between groups (*p* = 0.046). Although there was a tendency towards higher morphine doses in the block group, false discovery rate corrected comparisons at each time point revealed no statistically significant differences between groups.

**Fig. (4):**
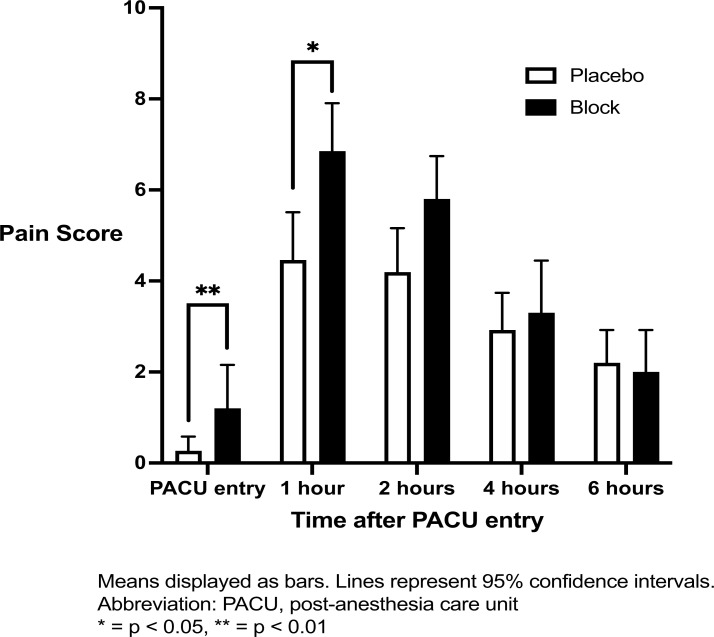
Pain score in the PACU over 6 hours.

Analysis of pain exposure over time (Fig. **4**) showed a significant difference between groups (p = 0.0021), with false discovery rate corrected comparisons at each time point showing a significant difference 1 hour after PACU entry and 2 hours after PACU entry. At 1 hour after PACU entry, the mean pain score was 7 (6 - 8) in the Block group and 4 (3 - 6) in the placebo group (q = 0.006). At 2 hours after PACU entry, the mean pain score was 6 (5 - 7) in the block group and 4 (3 - 5) in the placebo group (q = 0.03).

**Fig. (5):**
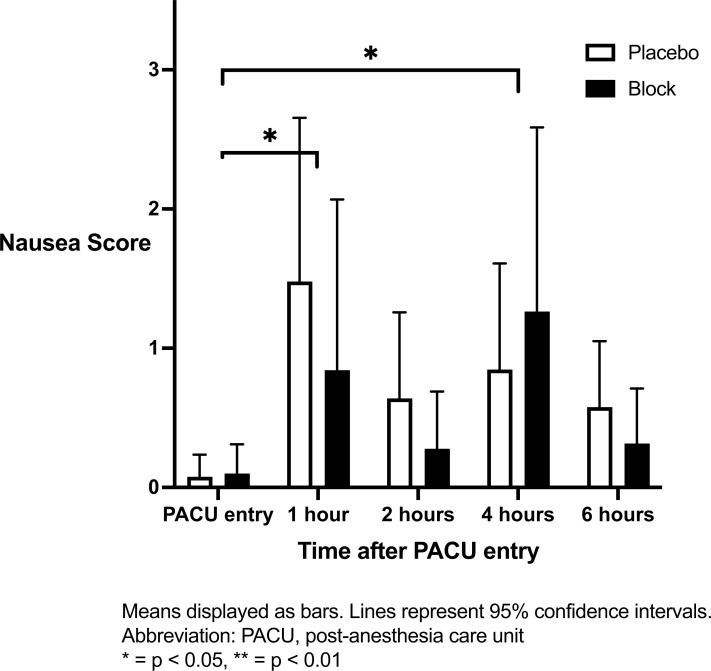
Nausea score in the PACU over 6 hours.

There was no significant difference between groups in the incidence of nausea over time (Fig. **5**). Examining both groups combined, compared to PACU arrival, patients experienced significantly more nausea at 1 hour after PACU entry (mean difference 1.1 [SE 0.^4^], q = 0.03) and at 4 hours after PACU entry (mean difference 0.9 [SE 0.^3^], q = 0.03).

The median PACU stay duration was 128 minutes (110 - 158 minutes) in the block group and 113 minutes (98 - 143 minutes) in the placebo group (*p* = 0.28).

There was no difference in either median time from surgery start to hospital discharge (Block group; 1540 minutes [1447 - 1811 minutes] *vs*. Placebo; 1703 minutes [1592 - 1821 minutes], *p* = 0.26), or median time from surgery start to last in-hospital narcotic dose (Block; 1355 minutes [733 - 1467 minutes] *vs*. Placebo; 698 minutes [422 - 1657 minutes], *p* = 0.15). Just prior to hospital discharge, but before the last dose of narcotics, the block group had significantly higher median pain scores (Block 2 [1 - 4] *vs*. Placebo 1 [0 - 2], *p* = 0.03).

## 5 DISCUSSION

Usually, prolonged hospital admissions following total endoscopic endonasal pituitary surgery result from headache and nausea +/- vomiting. The etiology of their postoperative headache is likely multifactorial, but one theory is that stimulation of the peripheral trigeminal (Cranial Nerve number V) fibers in V1 (ophthalmic branch) and V2 (maxillary branch) sensory distribution of the face by the surgical endoscope and surgical trauma may lead to extravasation and release of inflammatory mediators (Substance P (SP), calcitonin gene-related peptide (CGRP), and Vasoactive Intestinal Peptides (VIPs)) from the local area via the trigemino-vascular system. This “sterile neurogenic peri-vascular inflammation” can ultimately cause meningeal blood vessel vasodilation, increased cerebral blood flow, and migraine-type post-op headaches [2-4]. It has been shown that repetitive injection of local anesthetics in the distribution of the supra-orbital nerve (SON) V1 and infra-orbital nerve (ION) V2 can decrease the incidence of chronic idiopathic migraine headaches not related to surgery [5]. Thus, potentially local anesthetics injected in the distribution of the supra-orbital (V1) and infra-orbital (V2) nerves were postulated to pre-emptively prevent this sequence of events that ultimately lead to post-operative headache after trans-sphenoidal pituitary surgery.

**Fig. (6):**
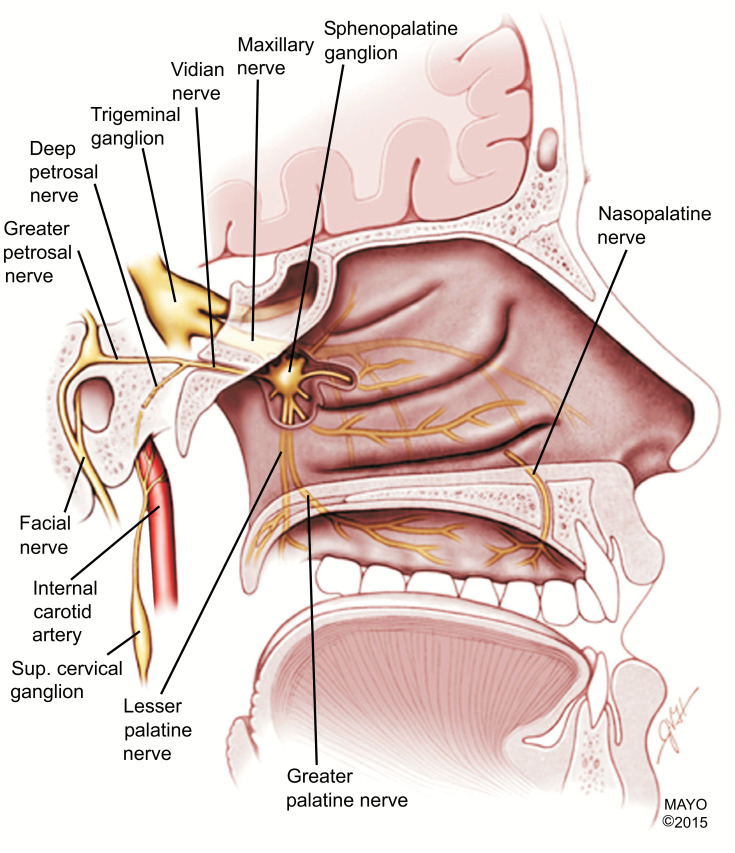
Sagittal view drawing through the nasopharynx demonstrates the relationship of the trigeminal ganglion with the sphenopalatine ganglion and its direct connections. Reprinted from: The Sphenopalatine Ganglion: Anatomy, Pathophysiology, and Therapeutic Targeting in Headache. Matthew S. Robbins *et al*. Headache Feb 2016; 56:240-258. With permission from Publisher John Wiley and Sons. **Note:** The sphenopalatine ganglion is associated with the trigeminal nerve, the major nerve involved in headache disorders. SPG is an extracranial parasympathetic ganglion located behind the nasal bony structures. It has two ganglia, one in each of the bilateral fossae located posterior to the middle turbinate. It is made up of 3 nerves: the sensory, sympathetic and parasympathetic.

**Fig. (7):**
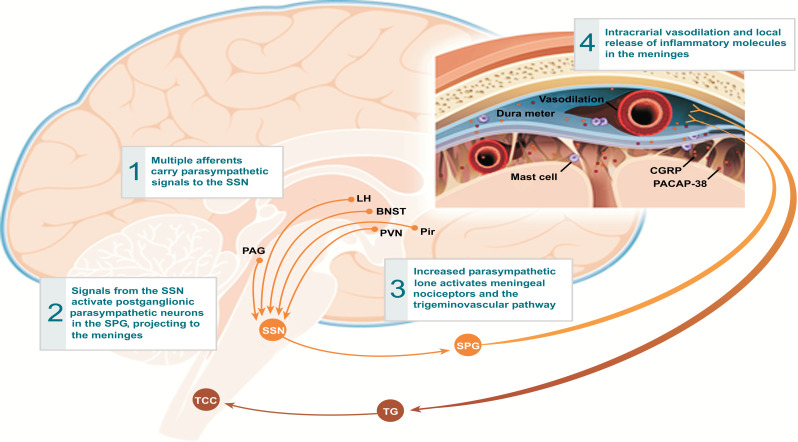
Activation of the Meningeal Receptors by increased Parasympathetic tone. Reprinted From: Supplement Article: “A Phase by Phase Review of Migraine Pathophysiology”. David W. Dodick. Headache: The Journal of Head and Face Pain. Headache 2018; 58:4-16. With Permission from publisher Wiley Periodicals, Inc. **Abbreviations:** BNST = Bed nucleus of stria terminalis; LH = Lateral hypothalamus; PAG = Periaqueductal gray; Pir = Piriform cortex; PVN = Paraventricular hypothalamic nucleus; SPG = Sphenopalatine ganglion; SSN = Superior salivatory nucleus; TCC = Trigeminal cervical complex; TG = Trigeminal ganglion.

**Fig. (8):**
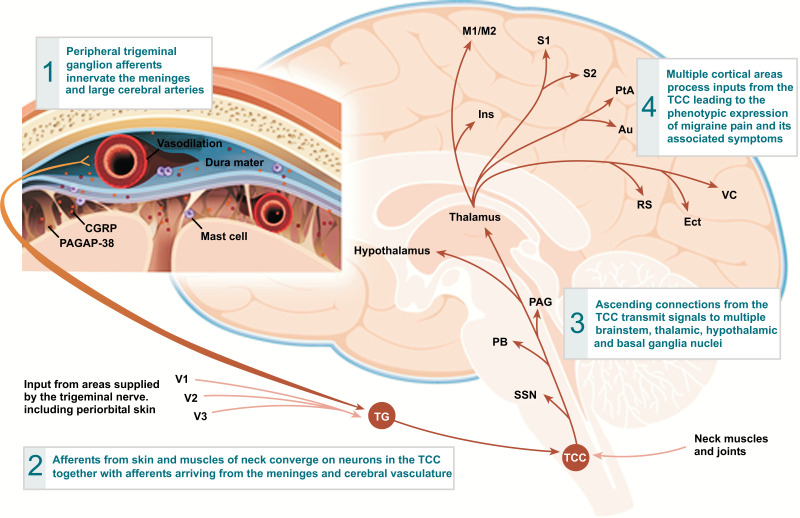
The Triggering of Pain *via* the Trigemino-vascular pathway. Reprinted From: Supplement Article: “A Phase by Phase Review of Migraine Pathophysiology”. David W. Dodick. Headache: The Journal of Head and Face Pain. Headache 2018; 58:4-16. With Permission from publisher Wiley Periodicals, Inc. **Abbreviations:** Au = Auditory; CGRP = Calcitonin gene related peptide, signaling neurotransmitter in the trigemino-vascular system; Ect = Entorhinal; Ins = Insula; M1/M2 = Motor cortices; PACAP-38 = Pituitary adenylate cyclase activating neuro-peptide-38 amino acids; PAG = Periaqueductal gray; PB = Parabrachial nucleus; PtA = Parietal associations; RS = Retrosplenial; S1/S2 = Somatosensory cortices; SSN = superior salivatory nucleus; TCC = Trigeminal cervical complex; TG = Trigeminal ganglion; VC = Visual cortices; V1 = Ophthalmic branch of the trigeminal nerve; V2 = Maxillary branch of the trigeminal nerve; V3 = Mandibular branch of the trigeminal nerve.

These study results showed that the addition of supra-orbital and infra-orbital sensory trigeminal nerve blocks intra-operatively to general anesthesia during transsphenoidal, total endo-nasal pituitary surgery does not appear to reduce postoperative headache and thus, 6-hour post-op morphine PCA consumption as compared to patients receiving only general anesthesia. In fact, the placebo group had less postoperative pain and morphine use, along with a shorter median PACU stay. As the standard deviations in both groups were varied, this effect was not clinically significant. Possible explanations for this unexpected pain and morphine consumption post-op response in the block group may include:

(1) It may be more difficult to accurately execute the supra-orbital V1 and infra-orbital nerve V2 blocks under general anesthesia than expected. The terminal sensory nerve anatomy of patients may vary, and so the block, placed using landmarks, may not actually capture all branches of the trigeminal nerves V1 and V2, which may then eventually trigger the trigeminovascular effects, leading to post-op headaches. As the patients were under general anesthesia, the presence/absence and extent of numbness were not evaluated prior to surgery. Post-op evaluation of this would have led to unblinding the patient and the data collection nurse, so this was not performed. Thus, the two groups showed similar postoperative pain exposure data and morphine use.

(2) It is possible that the administration of normal saline solution used in the placebo group may have some analgesic properties, resulting in similar pain exposure and 6-hour post-op morphine use data.

(3) Most surprisingly/unexpectedly, there was a tendency for slightly paradoxically increased pain exposure, morphine usage, and median time of PACU stay in the block group. One possible explanation for this could be the influence of another mechanism in addition to the sensitization of the trigeminal ganglion in the production of post-op headaches. The sphenopalatine ganglion (SPG), a primarily parasympathetic ganglion, located in the pterygopalatine fossa (posterior to the middle nasal turbinate), is known to communicate directly with the trigeminal ganglion via several branches of the infra-orbital or maxillary V2 nerve (Fig. **6**). Both the sphenopalatine and trigeminal nerve ganglia have been extensively studied in migraine [10-12]. Perhaps through some type of neuromodulation, the parasympathetic nervous system vasodilating effects of the SPG are enhanced if the trigeminal V1 and V2 sensory nerve blocks are performed. This may lead to more cerebral vasculature dilation and brain inflammatory mediator release (SP, CGRP, VIP), causing a slight increase in migraine-type post-op headache in the block group (Figs. **7** and **8**) [11]. A recent study [13] showed that bilateral SPG blocks as an adjuvant to general anesthesia for endoscopic trans-sphenoidal pituitary surgery might decrease post-op pain. Blocking the SPG is more.

(4) invasive and riskier [14] than the superficial trigeminal verve blocks of V1 and V2. Anesthetic pledgets, intranasal injections administered by surgeons, and specialized endoscopic guided catheters like the “SphenoCath” [12] performed by radiologists under fluoroscopy can be used to administer this SPG block.

(5) Perhaps future studies should focus on randomized, controlled data using both the trigeminalnerve blocks combined with the sphenopalatine ganglion block as a multimodal regional adjunct to general anesthesia for transsphenoidal pituitary resection.

(6) As there appeared to be little difference in pain exposure and morphine consumption post-op, no significant difference was reported in post-op nausea over time between the block and placebo groups.

(7) In addition to the pain complexity described, it is possible that patients may have had variable amounts of preoperative anxiety and associated sleep disturbance, which may variably amplify/ affect their perceived postoperative surgical pain experience. All patients received midazolam 1-2 mg IV pre-operatively as a premedication prior to surgery based on their subjective reported level of preoperative anxiety. A total of 81% of placebo (P) patients received 2 mg of midazolam IV, and 78% of block (B) patients received 2 mg of midazolam IV. We recorded baseline preoperative pain data prior to surgery, which was considered in our statistical calculations related to pain exposure. We did not gather data on sleep disturbance and anxiety.

The postoperative time to discharge and time to last dose of narcotics from the start of surgery for the block and placebo groups did not have statistically significant differences. Considering pain on discharge, the placebo group had less pain, although the final median pain scores of 1/10 for placebo and 2/10 for block did not seem to have clinical significance. However, examining the individual pain scores, the block group was found to have greater pain data, showing some patients with significant pain on discharge.

## CONCLUSION

In conclusion, the mechanism of the pain response to pituitary surgery via the total endo-nasal endoscopic technique appears to be more complex than expected. Likely, several nerves and ganglia interact via a complex mechanism of neuromodulation to determine the final post-op headache intensity experienced by the patient. Simple peripheral sensory nerve supra-orbital V1 and infra-orbital V2 nerve blocks, in addition to general anesthesia, do not decrease pain exposure or 6-hour post-op morphine PCA consumption compared to general anesthesia alone.

## Figures and Tables

**Table 1:** Demographic patient characteristics.

**-**	**Placebo (P) / Control** **Normal Saline**	**Block (B) 0.5%** **Ropivicaine with epi**	**All**	**Standardized** Difference*
n	26 (56.5%)	20 (43.4%)	46 (100%)	-
Age	47.9 ± 13.0	41.9 ± 11.7	45.2 ± 12.7	0.48
Gender	-	-	-	0.45
Female	9 (34.6%)	12 (60%)	21 (45.7%)	-
Male	17 (65.3%)	8 (40%)	25 (54.3%)	-
Height (cm)	173 ± 9	171 ± 11	172 ± 10	0.26
Weight (kg)	86.8 ± 15.6	90.8 ± 20.9	88.6 ± 18.1	0.21
BMI (kg/m^2^)	28.9 ± 4.2	31.6 ± 8.9	30.1 ± 6.8	0.39

**Note:** Data are mean ± SD, or n (%); *Reference: Cohen J., Statistical Power Analysis for the Behavioral Sciences, 2nd Edition, Hillsdale, NJ: Erlbaum; 1988.

## Data Availability

All data generated or analyzed during this study are included in this published article and its supplementary information file. All available raw data can be shared on reasonable request to the corresponding author [US].

## LIST OF ABBREVIATIONS

ASA: American Society of Anesthesiologists
BARF: Baxter Retching Faces
CGRP: Calcitonin Gene-Related Peptide
PACU: Post-Anesthesia Care Unit
PCA: Patient-Controlled Analgesia
SION: Supra-orbital and Infra-Orbital Nerve
SK: Single Surgeon
UCSF: University of California, San Francisco, USA
VIPs: Vasoactive Intestinal Peptides

## References

[1] Cousins M.J., Bridenbaugh P.O. (1998). Neural blockade in clinical anesthesia and management of pain. Neural blockade for pediatric surgery..

[2] Goadsby P.J., Edvinsson L. (1993). The trigeminovascular system and migraine: Studies characterizing cerebrovascular and neuropeptide changes seen in humans and cats.. Ann. Neurol..

[3] Lauritzen M. (1987). Cerebral blood flow in migraine and cortical spreading depression.. Acta Neurol. Scand. Suppl..

[4] Zagami A.S., Goadsby P.J., Edvinsson L. (1990). Stimulation of the superior sagittal sinus in the cat causes release of vasoactive peptides.. Neuropeptides.

[5] Ilhan Alp S., Alp R. (2013). Supraorbital and infraorbital nerve blockade in migraine patients: results of 6-month clinical follow-up.. Eur. Rev. Med. Pharmacol. Sci..

[6] Mariano E.R., Watson D., Loland V.J. (2009). Bilateral infraorbital nerve blocks decrease postoperative pain but do not reduce time to discharge following outpatient nasal surgery.. Can. J. Anaesth..

[7] McAdam D., Muro K., Suresh S. (2005). The use of infraorbital nerve block for postoperative pain control after transsphenoidal hypophysectomy.. Reg. Anesth. Pain Med..

[8] Michael T.L., Scott A.S., Robert A.S., Matthew J.J., Richard E. (1994). Comparison of intraoral and percutaneous approaches for infraorbital nerve block.. Acad. Emerg. Med..

[9] Brigid C.F., Edward C. (2006). Postoperative nausea and vomiting and pain after trans-sphenoidal surgery: A review of 877 patients.. Anesth. Analg..

[10] Matthew S.R., Robertson C.E., Kaplan E., Ailani J., Charleston L.I.V., Kuruvilla D. (2016). The sphenopalatine ganglion: Anatomy, pathophysiology, and therapeutic targeting in headache.. Headache.

[11] Dodick D.W. (2018). A phase-by-phase review of migraine pathophysiology.. Headache.

[12] Binfalah M., Alghawi E., Shosha E., Alhilly A., Bakhiet M. (2018). Sphenopalatine ganglion block for the treatment of acute migraine headache.. Pain Res. Treat..

[13] Ashgan R.A., Sameh A.S., Rahman A.S.M.A., Ashgan R.A. (2010). Bilateral sphenopalatine ganglion block as adjuvant to general anesthesia during endoscopic trans-nasal resection of pituitary adenoma.. Egypt. J. Anaesth..

[14] Hill J.N., Gershon N., Gargiulo P.O. (1983). Total spinal blockade during local anesthesia of the nasal passages.. Anesthesiology.

